# Water-Extracted *Prunella vulgaris* Alleviates Endometriosis by Reducing Aerobic Glycolysis

**DOI:** 10.3389/fphar.2022.872810

**Published:** 2022-04-04

**Authors:** Min Kyoung Cho, Ling Jin, Jung Ho Han, Jung-Suk Jin, Se-Yun Cheon, Su Shin, Sung-Jin Bae, Jang-Kyung Park, Ki-Tae Ha

**Affiliations:** ^1^ Korean Medical Research Center for Healthy Aging, Pusan National University, Yangsan, South Korea; ^2^ Department of Korean Medical Science, School of Korean Medicine, Pusan National University, Yangsan, South Korea; ^3^ Department of Anatomy, Kosin University College of Medicine, Busan, South Korea; ^4^ Department of Korean Obstetrics and Gynecology, Pusan National University Korean Medicine Hospital, Yangsan, South Korea

**Keywords:** Aerobic glycolysis, endometriosis, lactate dehydrogenase, pyruvate dehydrogenase kinase, *Prunella vulgaris*, ulsoric acid, Warburg-like metabolism

## Abstract

Endometriosis is a chronic inflammatory disorder caused by abnormal adhesion of endometrial tissue to the outside of the uterus. The combination of surgery, non-steroidal anti-inflammatory drugs, and hormone treatment is well established therapy for endometriosis, however, case reports have showed that high rates of relapse and unpleasant side effect. For these reasons, recently, the studies have been focused on the Warburg-like metabolic shift of endometriosis. *Prunella vulgaris* is one of traditionally used herbal medicine for inflammatory disease and the anti-estrogenic effects of *P. vulgaris* is well-established. Therefore, in this work, we evaluated water-extracted *P. vulgaris* (PV) as a potential treatment for endometriosis. To this, we artificially induced endometriosis in ovarectomized mice by intra-peritoneal inoculation of uterus extracts. PV was orally administered, and PV significantly alleviated endometriosis, particularly the growth of ectopic endometrial lesions in artificially endometriosis-induced mice. For the mechanism study of anti-endometriosis by PV, we designed an *in vitro* study using human normal endometrial stromal cells (T-HESCs) and human endometrial cell (12Z) obtained from patients with endometriosis. PV strongly induced the apoptosis of 12Z cells rather than T-HESCs by control the activity or expression of aerobic glycolysis enzymes, such as lactate dehydrogenase A (LDHA), pyruvate dehydrogenase A, and pyruvate dehydrogenase kinase 1/3. In addition, lactate production was enhanced, and oxygen consumption rate was suppressed in 12Z cells upon PV treatment. These changes in aerobic glycolysis eventually caused mitochondrial damage following decreased mitochondrial membrane potential and excessive mitochondrial ROS production. Especially, ulsolic acid (UA), one of the compounds in PV considerably led 12Z cell apoptosis with inhibition of LDHA activity. Therefore, UA could be a major active substance of PV in terms of endometriosis inhibitors. In conclusion, this study provides the evidence that the beneficial efficacy of PV for the prevention/treatment of endometriosis.

## Introduction

Endometriosis is a gynecological disorder characterized by abnormal growth of endometrial cells outside the uterus, commonly involving the ovaries, fallopian tubes, and the lining of the pelvic cavity (Malvezzi et al., 2020). Despite the high prevalence of the disease affecting up to 10% of reproductive-aged women, the pathogenesis of endometriosis is unclear (Zubrzycka et al., 2020; Arafah et al., 2021). The most well established hypothesis regarding the pathophysiology of endometriosis is retrograde menstruation. However, this hypothesis does not fully explain the mechanisms underlying endometriosis (Klemmt and Starzinski-Powitz, 2018; Chapron et al., 2019). Thus, several other mechanisms, such as inflammation, immunological dysregulation, hormone imbalance, and both genetic and epigenetic factors are thought to act in combination as the cause of endometriosis (Chapron et al., 2019; Zubrzycka et al., 2020).

Recently, a cancer-like glycolytic phenotype was reported in patients with endometriosis, as measured by metabolite and transcript levels (Marianna et al., 2017; Rytkonen et al., 2020; Zheng et al., 2021). Glucose transporter 1 (GLUT1), lactate dehydrogenase A (LDHA), and pyruvate dehydrogenase kinase 1 (PDK1) have been suggested as key enzymes for maintaining glycolytic metabolism in endometriosis (McKinnon et al., 2014; Young et al., 2014; Lee et al., 2018; Kido et al., 2020). LDHA is a key enzyme that converts pyruvate into lactate (Zhang et al., 2015; Urbanska and Orzechowski, 2019), while PDK1 is an enzyme responsible for inducing aerobic glycolysis by suppressing the activity of the pyruvate dehydrogenase complex by phosphorylating its E1α subunit (PDHA) (Stacpoole, 2017; Park et al., 2018). In addition, several studies have shown that targeting LDHA or PDK1 might be an effective non-hormonal strategy for the treatment of endometriosis by reducing cell survival and inducing apoptotic cell death (Young et al., 2016; Horne et al., 2019; Kim et al., 2021a).

In Eastern Asia, the herbal medicine is commonly used for the treatment of endometriosis and is generally used to alleviate pain, promote fertility, and prevent relapse (Flower et al., 2012). *Prunella vulgaris* L. is a perennial herb belonging to the family Labiatae and is widely distributed in northeast Asia, including China, Japan, and Korea (Bai et al., 2016; Wang et al., 2019). *P. vulgaris* is commonly used as a herbal medicine for headaches, dizziness, scrofula, goiter, and mastitis (Bai et al., 2016). *P. vulgaris* and its components have anti-cancer effects on several malignant tumors, such as gastric, colorectal, thyroid, breast, and uterine cancers, because it induces apoptotic cell death (Lin et al., 2013; Han et al., 2015; Lim et al., 2020; Lin et al., 2020; Yu et al., 2021). In addition, *P. vulgaris* and its active components were reported to regulate glucose metabolism by means of metabolites and enzymes in cancer cells as well as in a diabetic mouse model (Hwang et al., 2012; Wang et al., 2014; Raafat et al., 2016; Zhang et al., 2020). The expression of enzymes related to glucose metabolism, such as enolase 1 and M2-type pyruvate kinase, can be decreased with *P. vulgaris* treatment (Wang et al., 2014). Several studies have demonstrated that *P. vulgaris* and betulinic acid, one of its active ingredients, have an inhibitory effect on the growth of endometrial cells through anti-estrogenic activity (Huang et al., 2006; Collins et al., 2009; Xiang et al., 2020). However, the molecular mechanisms underlying the inhibition of endometriosis by *P. vulgaris* have not been fully elucidated.

Based on previous experimental evidences, we hypothesized that changes in glucose metabolism might be the underlying mechanism driving *P. vulgaris*-induced endometriosis reduction. Therefore, in this study, we investigated the effect of water-extracted *P. vulgaris* (PV) on endometriosis *in vivo* and *in vitro*. The results suggest that PV reduced endometriotic lesions by inducing apoptosis and that the suppression of aerobic glycolysis might be one of the underlying molecular mechanisms.

## Materials and Methods

### Preparation of *P. vulgaris* Extracts

PV was purchased from the Korea Plant Extract Bank at the Korea Research Institute of Bioscience and Biotechnology (Chungju-si, Chungchungbuk-do, South Korea). The extract was dissolved in phosphate buffered saline (PBS) for the *in vitro* enzyme activity assay along with dimethyl sulfoxide (Sigma-Aldrich Chemical Co., Louis, Mo, United States), before being diluted using culture media for cell line experiments, or with corn oil immediately before use in animal studies.

### Animal Care

Five-week-old female C57BL/6 mice were purchased from Orient Bio (Seongnam, South Korea) and were maintained in polycarbonate cages during the experimental period at an animal facility at the Institute for Laboratory Animals of Pusan National University. A 12 h day-night cycle, room temperature of 22 ± 2°C, and humidity of 50–60% were automatically maintained in the animal facility. Isoflurane and CO_2_ inhalation was used for anesthesia and sacrifice, respectively. All experimental procedures were approved by the Institutional Animal Care and Use Committee of Pusan National University (Pusan, Republic of Korea; PNU-2020-2679).

### Endometriosis Mouse Models

Endometriosis was induced based on a previous protocol (Choi et al., 2018) with slight modification. Briefly, six-week-old C57BL/6 female mice were ovarectomized and recuperated before estrogen injection. After 14 days, mice were subcutaneously injected with 100 mg/kg β-estradiol (Santa Cruz Biotechnology; Dallas, TX, United States) suspended in corn oil every week. The uteri of donor mice were isolated and chopped using Gentle Max (Miltenyi Biotec, Bergisch Gladbach, Germany) and intraperitoneally inoculated into recipient mice at a 1:1 ratio. One day after uterine injection, the mice were orally administered PV 5 days per a week, and 100 mg/kg β-estradiol was subcutaneously injected once a week for 3 weeks. The dosages of PV given in mice were calculated based on 50% growth inhibition concentration at 48 h after PV treatment in 12Z cells and these two dosages (0.820 and 4.100 mg/kg) were smaller than the dosages calculated from formula provided by FDA (Nair and Jacob, 2016). Three weeks after uterine injection, the mice were euthanized, and endometriotic lesions were excised from the surrounding tissue to evaluate their number and weight. The number of mice per group was five.

### Cell Culture

Immortalized human endometrial stromal cells (T-HESCs) and immortalized human endometriotic epithelial (12Z) cells were purchased from the American Type Culture Collection (ATCC; #CRL-4003, Rockville, MD, United States) and Applied Biological Materials (#T0764, Richmond, Canada), respectively. T-HESCs were maintained in phenol red-free DMEM/F12 (Welgene, Daegu, South Korea) supplemented with 10% inactivated charcoal (Sigma-Aldrich)-stripped fetal bovine serum (FBS, Invitrogen, Waltham, MA, United States), 1% ITS premix (BD Biosciences, San Jose, CA), and 1% penicillin/streptomycin (Gibco, Rockville, MD, United States). 12Z cells were maintained in DMEM/F12 (Welgene, Daegu, Korea) supplemented with 10% non-inactivated charcoal-stripped FBS and 1% penicillin/streptomycin (Thermo Fisher Scientific, Waltham, MA, United States). Both cell types were cultured at 37 °C in a humidified atmosphere containing 5% CO_2_.

### Cell Apoptosis Analysis

Cell proliferation and apoptosis were analyzed using 3-(4,5-dimethylthiazol-2-yl)-2,5-diphenyltetrazolium bromide (MTT) (Sigma) and propidium iodide (PI)/Annexin V (BD Biosciences, Franklin Lakes, NJ, United States) methods 24 h after PV treatment. Both 12Z cells and T-HESCs were plated onto 96 well at 1 × 10^4^ cells/well and treated with the indicated concentrations of PV. Cell viability was analyzed by measuring absorbance at 450 nm using a Spectramax M2 spectrofluorometer (Molecular Devices, San Jose, CA, United States). For PI/Annexin V detection, 3 × 10^5^ cells were plated to 6 wells and cell apoptosis was measured at excitation (Ex) 494/emission (Em) 525 nm for Annexin V and Ex 535/Em 617 nm for PI using Attune X (Thermo Fisher Scientific), and analysis was conducted using the FlowJo program (BD Bioscience).

### LDHA Activity Assay

The reduction of NADH (β-nicotinamide adenine dinucleotide disodium salt hydrate; reduced form, Tokyo Chemical Industry, Tokyo, Japan) was used to validate the LDHA activity of PV. One unit of LDH activity is defined as the amount of enzyme that catalyzes the conversion of lactate into pyruvate to generate 1.0 μM of NADH per minute at 37°C. Briefly, various concentrations of PV were incubated in buffer containing 2 mM pyruvate, 20 μM NADH, 20 mM HEPES-K^+^ (pH 7.2), and 10 ng of purified recombinant human LDHA protein for 20 min. The fluorescence of NADH was measured at Ex 340/Em 460 nm wavelength using a spectrofluorometer (Spectramax M2; Molecular Devices, Sunnyvale, CA, United States).

### Immunoblot Analysis

For protein analysis, total proteins were extracted using RIPA buffer and 1% NP-40 lysis buffer containing a protease inhibitor cocktail (Roche, Basel, Switzerland). The extracted proteins were quantified using the Bradford assay and subjected to immunoblot analysis. Briefly, the proteins were separated using SDS-PAGE and transferred to a PVDF membrane (Amersham Bioscience, Uppsala, Sweden). The first antibody, anti-human PARP (#9542s; Cell Signalling), Caspase-3 (#9665s, Cell Signalling), Caspase-9 (#9508s; Cell Signalling), Bax (NB100-56095; Novusbio), Bcl-2 (NB100-56098; Novusbio), PDHA (sc-377092; Santa Cruz), phospho-PDHA (ab177461; Abcam), LDHA (ab84716; Abcam), PDK1 (ADI-KAP-PK112; Enzo), PDK3 (ab182574, Abcam), and GAPDH (sc-32233; Santa Cruz). HRP-conjugated anti-rabbit IgG or anti-rat IgG (all from Invitrogen) were used as secondary antibodies. The specific bands were developed using a western blot detection kit (Bio-Rad, Hercules, CA, United States) using the ImageQuant LAS 4000 chemiluminescence imaging system (GE Healthcare, Munich, Germany).

### Metabolic Assays

The oxygen consumption rate and lactate production in 12Z cells were measured after 4 h of PV treatment to clarify the influence of PV on metabolic activity. First, the oxygen consumption rate was analyzed using an oxygen consumption rate assay kit (Abcam, Cambridge, MA, United States). 12Z cells were seeded in a black 96-well microplate at a density of 3 × 10^4^ cells. The next day, various concentrations of PV (0, 62.5, 125, and 250 μg/ml) were added, and the experiment was conducted according to the manufacturer’s instructions. Second, lactate production was measured using a lactate fluorometric assay kit (Biovision, CA, United States). After 4 h of PV treatment, the media was replaced with phenol red- and serum-free DMEM and incubated for 1 h at 37°C. Then, 1 μl of the medium from each well was assessed by fluorescence at Em 535/Ex 590 nm using the Spectramax M2 spectrofluorometer. Dichloroacetate (DCA) was used as a positive control for lactate production.

### Measurement of Mitochondrial Membrane Potential and Mitochondrial Reactive Oxygen Species

The MMP and mitoROS were analyzed using the cationic and lipophilic fluorescent dye, Tetramethylrhodamine (TMRM), and red mitochondrial superoxide indicator, MitoSOX™ (Invitrogen, Eugene, OR, United States), respectively. The 12Z cells were stained with TMRM 18 h after PV treatment using flow cytometry. After pre-treatment with PV for 4 h, 12Z cells were washed twice with pre-warmed PBS for TMRM staining or Hank’s balanced salt solution for MitoSOX staining. To measure MMP, TMRM was added at a final concentration of 20 nM and incubated for 30 min at 37°C. To assess mitoROS, 5 μM MitoSOX was added and incubated for 10 min at 37°C. After staining, 12Z cells were washed three times, and then the intensity of fluorescence was analyzed using Attune X (Thermo Fisher Scientific) and the geometric mean fluorescence intensity was calculated using the FlowJo program (BD Bioscience).

### High Performance Liquid Chromatography Analysis of PV Extract

Oleanolic acid (OA) (99% purity) and rosmarinic acid (RA) (98% purity) were purchased from Sigma-Aldrich, and ursolic acid (UA) (92.84% purity) was purchased from the Resource Bank of Korean Medicine, National Institute for Korean Medicine Development. The compounds were dissolved in methanol at a concentration of 10 mM for use as controls, according to the methods described in previous studies (Qiang et al., 2011; Chen et al., 2012). The PV extract was dissolved to 3.12 mg/ml, sonicated using ultrasound for 20 min, and filtered through a 0.45 μm syringe filter (Millipore Sigma, Burlington, MA, United States). HPLC analysis was conducted on an Agilent 1200 series system (Agilent Technologies, Santa Clara, CA, United States) equipped with a Shiseido UG120 column (5 μm, 6.4 × 250 mm) maintained at 25°C. The mobile phase was methanol (90%): 0.5% ammonium acetate (10%) for OA, UA, and methanol (52%), and 0.01% phosphoric acid (48%) for RA. Samples (20 μl) were analyzed at a flow rate of 0.6 ml/min using an ultraviolet detector at 210 nm for OA and UA, and at 310 nm for RA.

### Statistical Analysis

Values are expressed as the mean ± SEM. A two-tailed Student’s t-test was used for comparisons of two different groups and one-way ANOVA followed by Tukey’s post-hoc test was used for comparisons of multiple groups. These were performed using GraphPad Prism Software (GraphPad, San Diego, CA, United States).

## Results

### PV Inhibits the Growth of Endometriotic Tissue in Mice

**FIGURE 1:**
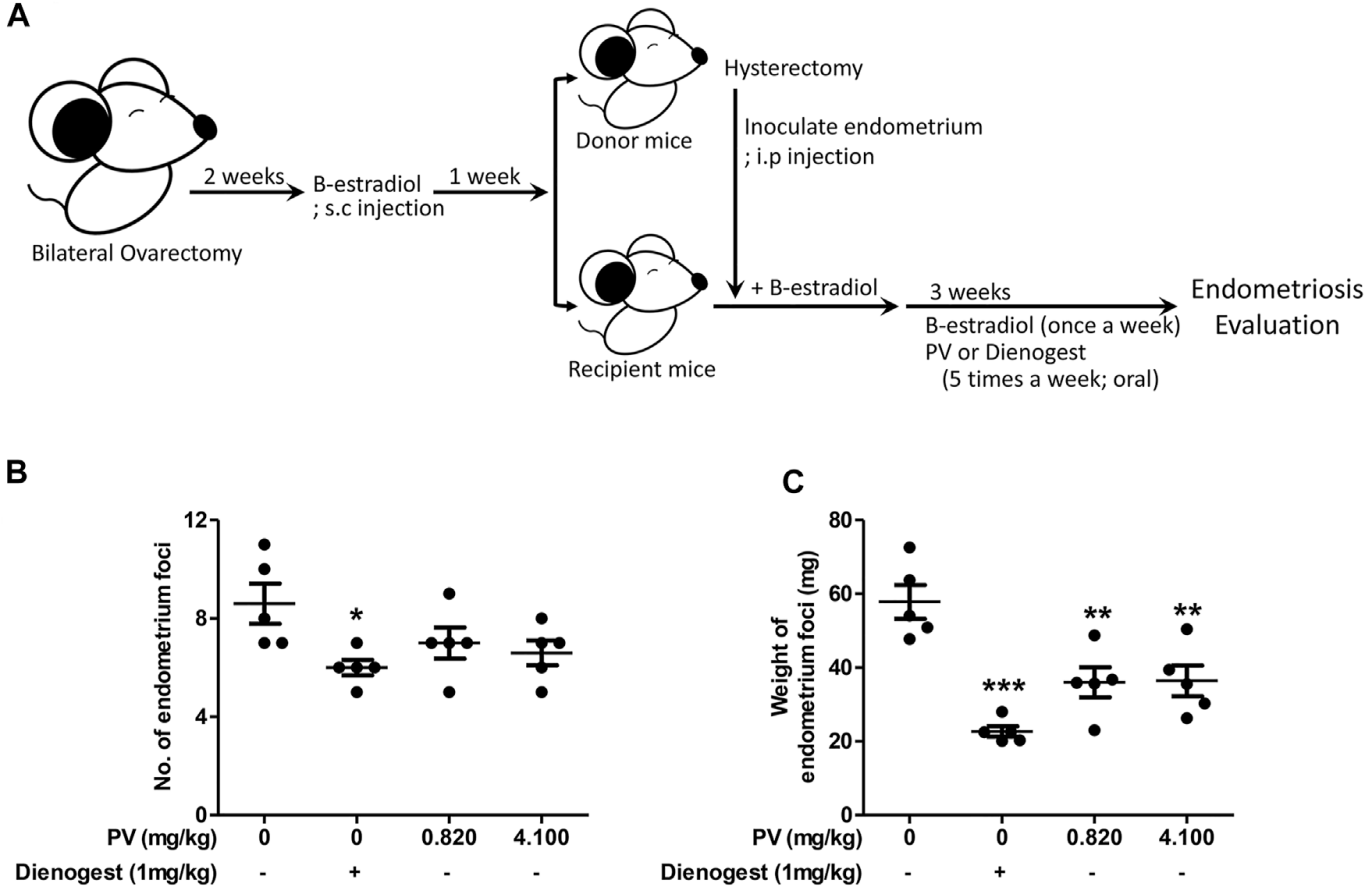
PV extract suppresses the growth of ectopic endometrial lesions in mice. The schematic of the estrogen mediated mouse endometriosis model and of PV treatment **(A)**. The number and weight of ectopic endometrial tissues **(B,C)**. Data are expressed as mean ± SEM. Statistical analysis was conducted using a *t*-test (*; *p* < 0.05, **; *p* < 0.01).

To evaluate if PV is beneficial for people with endometriosis, we first applied PV to a mouse endometriosis model. PV extract was administered orally at doses of 0.820 mg/kg and 4.100 mg/kg body weight or with 1 mg/ml of dienogest five times a week for 3 weeks after intraperitoneal inoculation of donor uterus tissue (Figure 1A). Dienogest, the first-line drug for endometriosis (Andres Mde et al., 2015; Liu et al., 2021), was used as a control to evaluate the efficiency of PV in endometriosis. The PV group showed a significant reduction in cyst-like ectopic lesion volume compared to the untreated mice group. However, a significant reduction in the volume and number of endometriotic lesions was observed in the dienogest-treated mouse group (Figures 1B,C). In addition, to clarify the safety of PV, kidney and liver tissues were examined using H&E staining. During histological examination, the doses of PV used in the *in vivo* experiment were deemed non-toxic (Supplementary Figure S1). These results indicate that the growth of ectopic endometriotic tissues can be ameliorated by oral administration of PV.

### PV is Effective at Inducing the Apoptosis of Human Endometriotic Epithelial Cells

**FIGURE 2:**
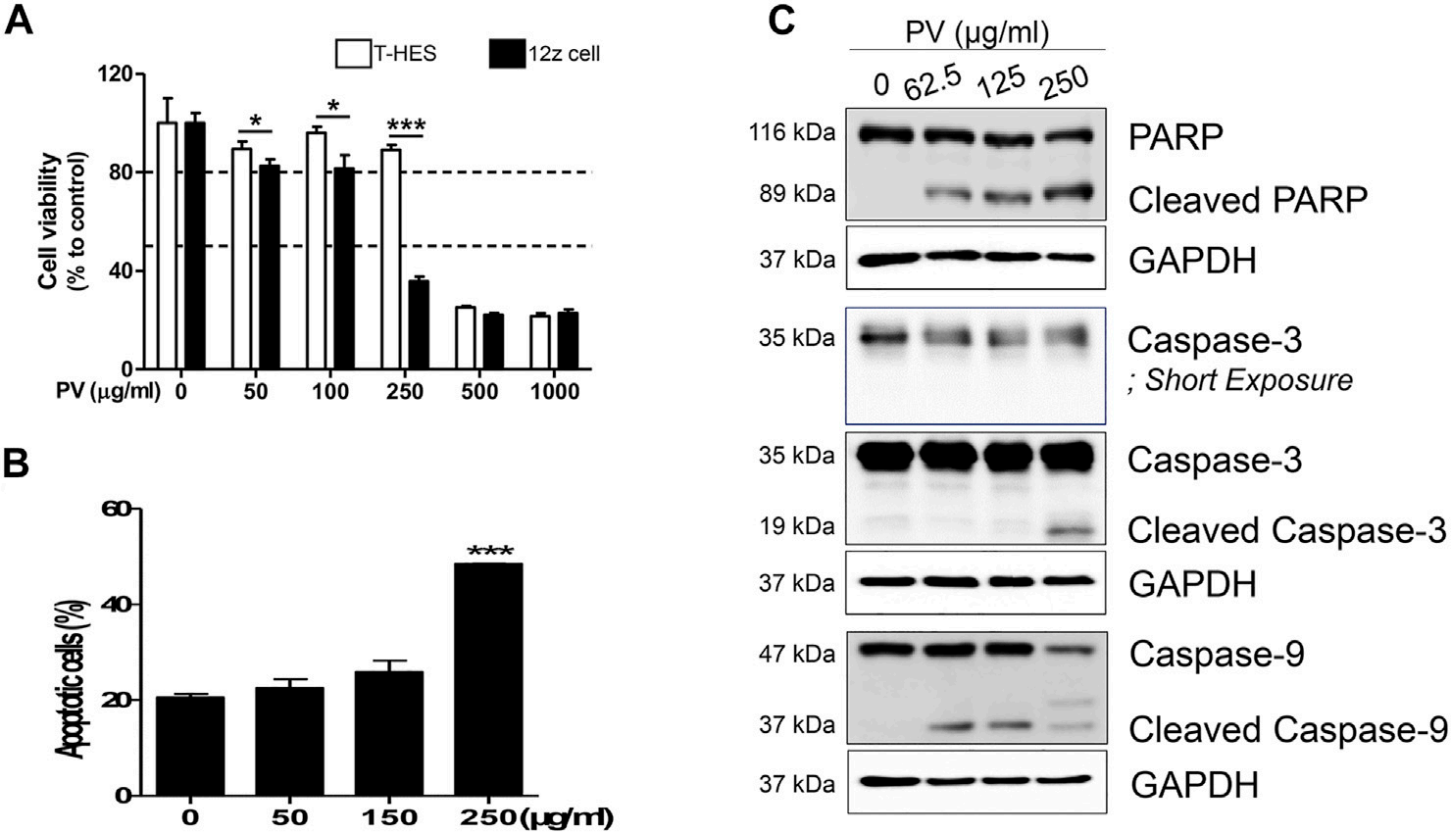
PV induces human endometriotic cell death. Increasing concentrations (0–1000 μg/ml) of PV were administered to human endometriotic epithelial (12Z) cells and normal human endometrial epithelial cells (T-HESCs). After 24 h, the viability of both cells were analyzed using MTT **(A)** and the viability of 12Z cells was analyzed using Annexin V/PI stain **(B)**. The intracellular apoptosis signaling proteins in 12Z cells were detected using western blotting. As the control, GAPDH was present **(C)**. The specific molecular weight standards are represented on the left. Data are expressed as mean ± SEM. Statistical analysis was conducted with the Tukey one-way ANOVA and *t*-test (*; *p* < 0.05, ***; *p* < 0.001).

To determine the inhibitory mechanism of endometriotic tissue growth in mouse experiments (Figure 1C), we first assessed the apoptosis of 12Z cells and T-HESCs after treatment with increasing doses of PV (0, 50, 100, 250, 500, and 1000 μg/ml) for 24 h. The colorimetric cell viability assay showed that PV induced cell death in both 12Z and T-HESCs in a dose-dependent manner, but the 12Z cells (50% growth inhibition concentration (GI_50_) 210.6 μg/ml) were more susceptible to PV-induced cell death than T-HESCs (GI_50_ 453.9 μg/ml) (Figure 2A). Similar to the colorimetric cell viability assay, the percentage of apoptotic cells, including necroptic, early, and late apoptotic cells, started to increase at a concentration of 150 μg/ml, and these apoptotic cell populations were dramatically increased at a concentration of 250 μg/ml in 12Z cells (Figure 2B and Supplementary Figure S2).

Apoptosis occurred through a signaling cascade; therefore, we next analyzed the changes in the intracellular apoptosis signaling pathway in 12Z cells by western blotting. 12Z cells were exposed to various concentrations of PV, and the protein levels and cleaved forms of caspase-3 and -9 and PARP were examined. PV induced activation of procaspase-9 into cleaved caspase-9 and cleavage of procaspase-3 into its active form. PARP, downstream of the activation of caspase-3, is a well-known characteristic of apoptosis, and cleavage of the PARP protein was also detected in a dose-dependent manner (Figure 2C). These data suggest that PV effectively induces 12Z cell apoptosis by activating the caspase-3 and -9 and PARP pathways, indicating the activation of the mitochondrial pathway for apoptosis.

### PV-Led 12Z Cell Apoptosis Occurs Through the Mitochondrial Apoptotic Signal Pathway

Mitochondria play an important role in the initiation and regulation of apoptosis. The mitochondrial-dependent apoptosis pathway involves changes in MMP, mitoROS generation, and ATP synthesis by oxidative phosphorylation, which is a phenomenon of mitochondria disruption (Nicholls, 2004; Martinez-Reyes and Chandel, 2020). The change in the mitochondrial membrane potential causes mitochondrial outer membrane permeabilization, resulting in caspase-dependent cell death following the release of cytochrome C into the cytosol (Li et al., 1997). Therefore, membrane depolarization and excessive production of mitoROS are good indicators of mitochondrial dysfunction (Abraham et al., 2008; Dykens et al., 2008).

**FIGURE 3:**
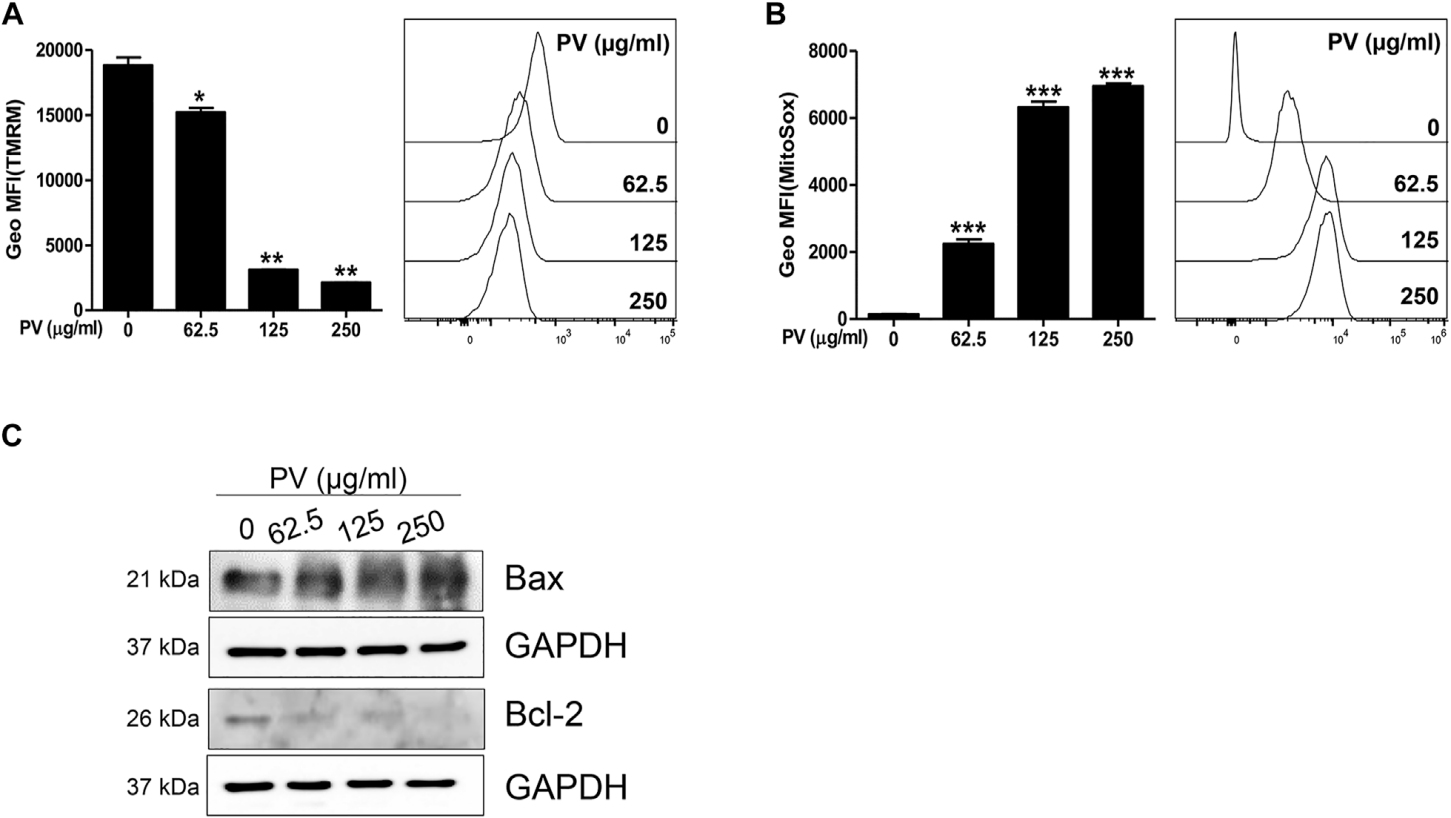
Mitochondria dysregulation occurs due to PV administration. The 12Z cells were stimulated with PV for 18 h and then stained with TMRM or MitoSox dye. The mitochondrial membrane potential and mitochondrial reactive oxygen species were assessed using flow cytometry **(A,B)**. Mitochondrial-dependent apoptosis signaling molecules, Bax and Bcl-2 were detected 24 h after PV treatment using western blotting **(C)**. The specific molecular weight is represented on the left. Data are expressed as mean ± SEM. Statistical analysis was conducted using a Dunnet one-way ANOVA (*; *p* < 0.05, **; *p* < 0.01, ***; *p* < 0.001).

Consistent with Figure 2C, we attempted to clarify whether the mitochondrial apoptotic pathway is a major factor in PV-induced apoptosis. First, we monitored both MMP and mitoROS using TMRM and Mitosox staining-based assays, respectively. The results showed that MMP was markedly reduced but mitoROS dramatically increased after 18 h of treatment with PV extract (Figures 3A,B). Because a loss of MMP promotes the release of cytochrome c from the mitochondria to the cytosol, we next examined the protein levels of Bax and Bcl-2, which are pro- and anti-apoptotic Bcl-2 members, respectively (Salah-Eldin et al., 2003). As shown in Figure 3C, the expression of Bax protein was increased, whereas the expression of Bcl-2 protein decreased upon PV treatment in 12Z cells. The increased ratio of Bax/Bcl-2 implies that PV favors the occurrence of apoptosis in 12Z cells. These results indicate that PV induces apoptosis of 12Z cells through the Bcl-2/Bax balance and ROS-mediated mitochondrial damage.

### PV Disrupts the Glycolytic Metabolism in Mitochondria Dependent on the Apoptosis Pathway

**FIGURE 4:**
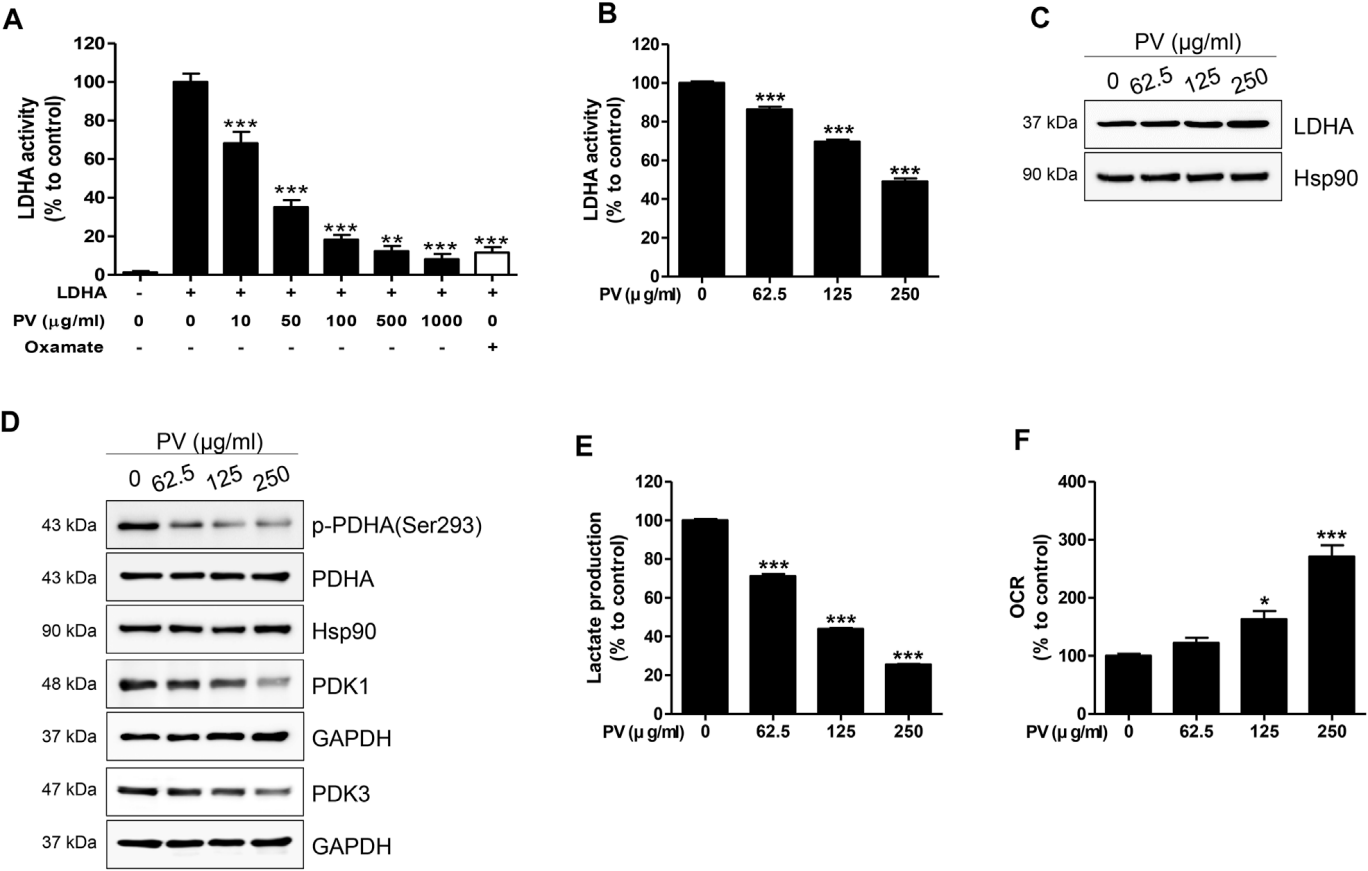
PV drives disruption of Warburg-like aerobic glycolysis. Solid *in vitro* LDHA enzymatic activity was estimated using recombinant human LDHA with the indicated concentrations of PV (0, 10, 50, 100, 500 and 1000 μg/ml). Oxamate (50 mM) was used as a positive control. To evaluate intracellular LDHA activity, 12Z cells were treated with various concentrations of PV (0–250 μg/ml) for 4 h. LDHA activity was measured using two independent methods of colorimetric observance at 340/460 nm **(A,B)**. Total forms of LDHA, PDHA, PDK1, PDK3, and phosphorylated PDHA (Ser293) were detected using western blotting. Hsp90 and GAPDH were used as normalization controls **(C,D)**. The lactate production and oxygen consumption rate in 12Z cells was measured after increasing doses of PV treatment. Oxygen consumption rate was measured in black 96-well microplates and lactate was quantified in phenol red and serum free DMEM by fluorescence at 535/590 nm **(E,F)**. Data are presented as mean ± SEM. Statistical analysis was conducted using a Dunnet one-way ANOVA (*; *p* < 0.05, **; *p* < 0.01, ***; *p* < 0.001).

In glycolytic cells, excessive pyruvate influx into the mitochondria leads to an accelerated tricarboxylic acid cycle, resulting in impaired mitochondria (Martinez-Reyes and Chandel, 2020). As shown in Figure 3, PV could lead to mitochondrial malfunction, so we next determined the influence of PV on glycolysis in 12Z cells. Therefore, we measured the activity and expression of LDHA, PDHA and PDK1/3. As shown in Figures 4A–C, PV significantly suppresses the *in vitro* enzymatic and intracellular LDHA activity, while the expression of LDHA did not change. In addition, PV markedly inhibited the expression of PDK1/3 and phosphorylation of PDHA (Figure 4D). These data imply that the mitochondrial apoptosis signal induced by PV is associated with enhanced PDH and suppressed LDHA activity. As LDHA and PDH are essential enzymes for the conversion of pyruvate into lactate or acetyl-CoA, respectively (Koukourakis et al., 2005; Zhang et al., 2015; Urbanska and Orzechowski, 2019), we next analyzed the lactate production and oxygen consumption rate after 4 h of PV treatment. The results showed that PV significantly decreased the amount of extracellular lactate but increased oxygen consumption in a dose-dependent manner (Figures 4E,F). Taken together, these results indicate that PV leads to 12Z cell apoptosis through the mitochondrial apoptosis signaling pathway, particularly glycolytic metabolism.

### UA is the Most Important Compound in PV-Led Cell Apoptosis

**FIGURE 5:**
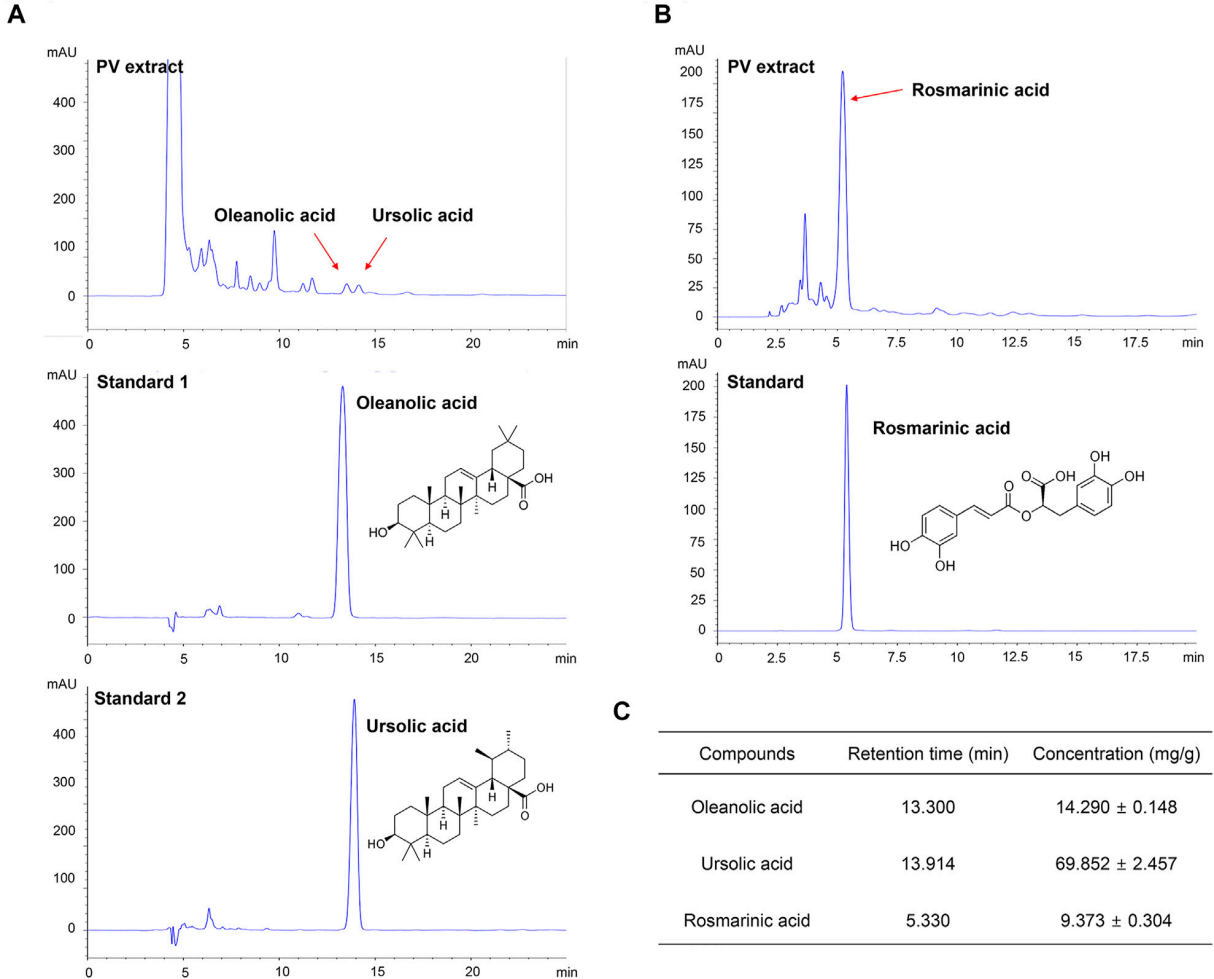
PV extract was analyzed using high performance liquid chromatography. The PV extract was sonicated with ultrasound for 20 min, and then filtered through a 0.45 μm syringe filter. As standard, methanol-dissolved oleanolic acid, rosmarinic acid, and ursolic acid were used. The samples were injected with 20 μl and analyzed at a flow rate of 0.6 ml/min using an ultraviolet detector at 210 nm for oleanolic acid and ursolic acid **(A)** and 310 nm for rosmarinic acid **(B)**. The concentration of each compound within the PV extract is summarized **(C)**.

To validate the purity of the PV extract used in this study, high-performance liquid chromatography analysis was performed. As shown in Figure 5, the PV extract was well separated within 15 min. OA, UA, and RA are well known as the main active substances in PV extract (Cheung and Zhang, 2008; Wang et al., 2019). Therefore, we estimated three substances by the retention times of the peaks with those of three standard solutions for OA, UA, and RA under the same conditions. To evaluate the reproducibility of the extraction efficiency, the extraction was repeated three times using the same sample. The results showed that the contents of OA, UA, and RA in 1 g of PV were 14.290 ± 0.148 mg, 69.852 ± 2.457 mg, and 9.373 ± 0.304 mg, respectively (Figure 5C). Based on GI_50_ of PV in 12Z cells, the amount of OA, UA, and RA were 6.72 μM (3.10 μg/ml), 33.14 μM (15.16 μg/ml), and 5.42 uM (1.95 μg/ml), respectively. This result indicated that UA was the most dominant compound in PV among the three substances.

**FIGURE 6:**
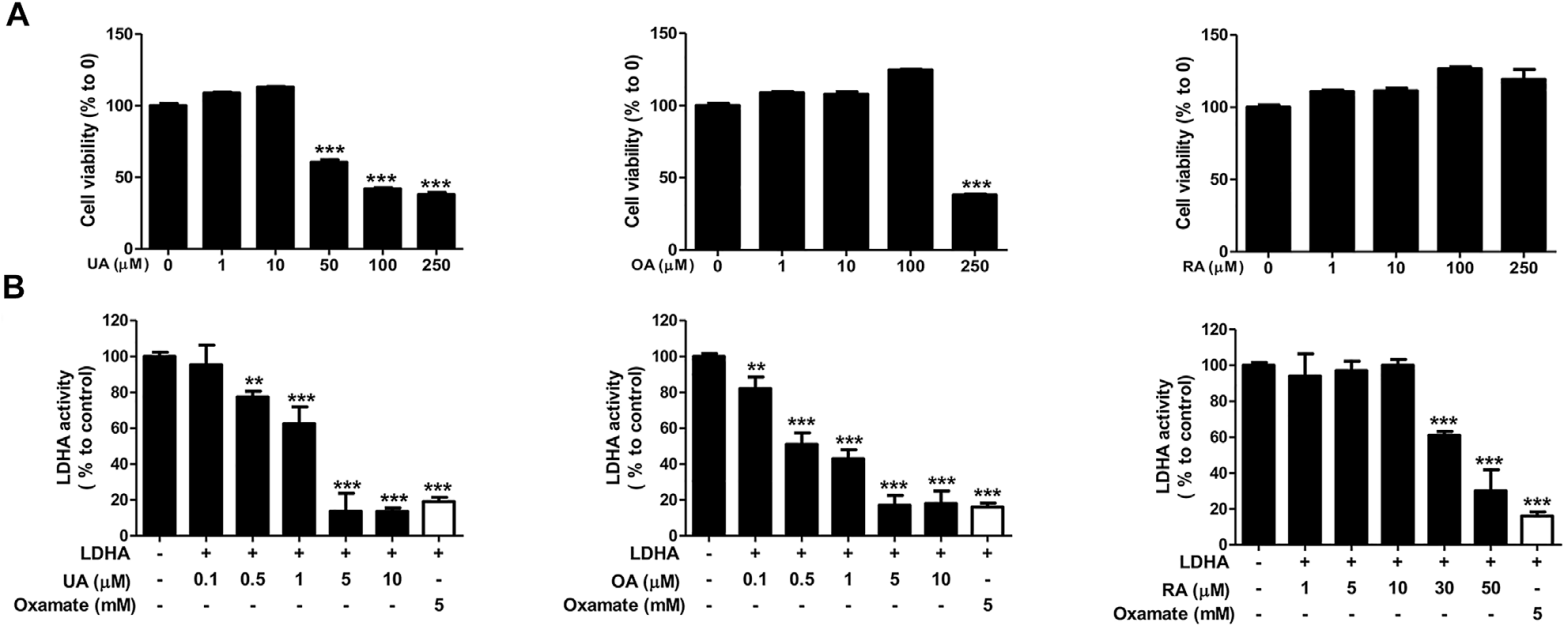
Ursolic acid is one of the main active ingredients in PV extract. Cell cytotoxicity of ursolic acid, oleanolic acid, and rosmarinic acid were analyzed after 24 h of treatment by MTT assay **(A)**. For detection of solid LDHA activity, the changes to NADH were measured. The indicated concentration of each substance was incubated with reaction buffer for 20 min. Fluorescence of NADH was detected at 340/460 nm using a spectrofluorometer. For a positive control, we used 6 mM oxamate **(B)**. Data are presented as mean ± SEM. Statistical analysis was conducted using a *t*-test (*; *p* < 0.05, **; *p* < 0.01, ***; *p* < 0.001).

We next examined the effect of each substance on cell viability and the LDHA activity of each substance to determine the main active compounds in PV-led 12Z cell death in terms of dysregulated glycolysis. In the colorimetric assay, RA did not induce cell apoptosis even at a dose of 250 μM and showed the smallest effect on LDHA activity (IC_50_ 42.93 μM) among the three substances. In contrast to RA, UA and OA induced apoptosis of 12Z cells (GI_50_ 87.58 and 218.6 μμM, respectively) and showed significant effects on LDHA activity (IC_50_ 1.76 and 0.64 μμM, respectively) (Figure 6). Because the content of OA and UA in 24.84 μg/ml of PV (IC_50_) were 0.79 and 3.91 μμM, respectively, it can be deduced that UA is the most effective substance in PV for inducing 12Z cell apoptosis via inhibition of LDHA activity among the three compounds.

## Discussion

Endometriosis is a major cause of female infertility, resulting from reduced oocyte quality and implantation failure (Orazov et al., 2019; Radzinsky et al., 2019). The current standard treatment consists of surgery and hormone treatment, however, there is a high rate of relapse and unpleasant side effects (Taylor et al., 2021). For these reasons, recent studies have been conducted to evaluate the use of traditional herbal medicines for endometriosis. The mechanisms underlying endometriosis have not been fully elucidated; however, cancer-like aerobic glycolysis has recently been considered as an important mediator for uncontrolled cell growth and resistance to apoptosis of endometriotic and malignant cancer cells (Doherty and Cleveland, 2013; Young et al., 2014; Marianna et al., 2017; Lee et al., 2019; Rytkonen et al., 2020; Kim et al., 2021b; Zheng et al., 2021). Recently, dichloroacetate (DCA), a well-known PDK inhibitor, was reported to reduce lesion size by inhibiting the glycolytic phenotype in a mouse model (Horne et al., 2019; Lee et al., 2019). Therefore, we hypothesized that the suppression of Warburg-like metabolism could be an alternative therapeutic target for endometriosis.

For the first time, we found suppressed growth of endometrial lesions by oral administration of PV in artificially induced endometriosis mice. We confirmed that PV efficiently induces mitochondria-mediated endometriotic cell apoptosis by disrupting mitochondrial function and abnormally decreasing aerobic glycolysis. PV has a greater impact on 12Z cells than on T-HESCs, it induces the activation of caspases 3 and 9, and regulates the Bcl-2 family following decreased MMP and sharply increased mitoROS. Furthermore, related to mitochondrial dysfunction, we found that PV could suppress the expression of PDK1/3 protein, phosphorylation of PDHA, and activity of LDHA in 12Z cells. Additionally, further *in vitro* experiments supported that UA is one of the main active components, in terms of LDHA inhibition, in the PV extract. Although the IC_50_ of OA for LDHA activity is more than twice that of UA, OA hardly induced the cytotoxicity in 12Z cells. In particular, the IC_50_ and GI_50_ of UA are 1.76 μM (correspond to 0.803 μg/ml) and 87.58 μM (correspond to 40 μg/ml), respectively. As the concentration of UA is about 7% of crude PV, the IC_50_ of solid *in vitro* LDHA activity is sufficient to explain PV-led inhibition of LDHA activity, but GI_50_ is not enough to compensate the cytotoxicity of PV in 12Z cells. Therefore, the efficacy of PV in endometriotic cell death accompanied by dysregulated aerobic glycolysis and mitochondrial function is not mediated by UA alone. Therefore, to discover a single substance associated with the anti-endometriosis effect of PV, further analysis of other compounds and in-depth activity studies are required.

In the regulation of both aerobic and anaerobic glycolysis, hypoxia-inducible factor (HIF) -1α is a key regulator (Lum et al., 2007; Del Rey et al., 2017). The activation of HIF-1α promotes the expression of the glycolytic enzymes PDK1 and PDK3 (Kim et al., 2006; Papandreou et al., 2006; Del Rey et al., 2017). HIF-1α induced PDK1 can change glucose metabolism from glucose oxidation to glycolysis by increasing OCR (Semba et al., 2016). Inhibition of PDK3 activity or expression levels increases mitoROS, oxidative phosphorylation, and pyruvate dehydrogenase activity under normoxic conditions (Kluza et al., 2012). Other pharmacology and molecular docking analyses have revealed that *P. vulgaris* (in both plant and prepared medicine forms) affects HIF-1α and the apoptosis signaling pathway in sleep disorders (Guo et al., 2020), thyroiditis (Shen et al., 2020), and diabetes mellitus (Jiao et al., 2021). Moreover, UA and its derivatives have been trialed as HIF-1α inhibitors with anticancer potential (Shan et al., 2016; Wang et al., 2016; Chi et al., 2017). Another study showed that RA affects the glycolytic pathway by suppressing glucose uptake, lactate production, and inhibiting HIF-1α expression (Han et al., 2015). Based on these reports, we can infer that inhibited aerobic glycolysis and mitochondrial-dependent cell apoptosis might be triggered by HIF-1α-PDK1/3 axis signaling.

The estrogen-related receptors (ERRs) are another possible factor for controlling pyruvate metabolism, resulting in the stimulated transcription of PDK2 and PDK4 (Zhang et al., 2006; Jeong et al., 2012). Several studies have suggested that *P. vulgaris* possesses estrogen resistant properties (Huang et al., 2006; Collins et al., 2009; Kim et al., 2014; Xiang et al., 2020). However, in our study, we observed that PV regulates the expression of PDK1/3 and activity of LDHA beside of anti-estrogenic effect. One possible reason is the different compositions of active ingredients in the extracts of *P. vulgaris* that were used in the present study versus those used in previous studies. We used the hydrothermal water extraction method so that the PV extracts were mainly hydrophilic, while others investigated the characteristics of ethanol- or methanol-extracted *P. vulgaris*, which is hydrophobic. Natural products are commonly obtained by hydrothermal extraction; therefore, our results are more widely applicable to the clinical field of traditional herbal medicine.

Importantly, our results demonstrate that a first-line drug for endometriosis, dienogest (Andres Mde et al., 2015; Mori et al., 2015; Liu et al., 2021), is more efficient than the suppressive effect of PV in inhibiting the incidence of endometriosis. However, with regard to side effects, several clinical studies have reported that dienogest causes abnormal vaginal bleeding, weight gain, headaches, depressed mood, and dizziness (Cosson et al., 2002; Uludag et al., 2021). *P. vulgaris* has been widely used in the clinical treatment of diabetes, hypertension, thyroiditis, and cancer (Yang et al., 2007; Zhao et al., 2018; Li et al., 2019; Wang et al., 2019), and it can administered cooked into food, as a pill, or as an extract. Although the side effects of PV have not been investigated, clinical research on PV is in the early stages and no research has been conducted on PV in children or in pregnant or breastfeeding women. Therefore, further studies in both animals and humans are needed to understand its benefits and downsides and to enable the safe use of PV for patients with endometriosis. Moreover, although we elucidated the notable anti-endometriosis effects of PV, the mode-of-action (MOA) of a single compound contained in PV-antagonized LDHA activity is unclear. Investigation of possible MOA for the active ingredients in PV using enzyme kinetics, protein-chemical binding assay, and *in silico* prediction or X-ray crystallographic analysis of protein-drug interaction should be conducted in the future. Understanding the MOA of single compounds of PV provides a resource for clinical trials of PV or single compounds in endometriosis. Furthermore, this may further suggest a new clinical strategy by presenting new targets for endometriosis.

**FIGURE 7:**
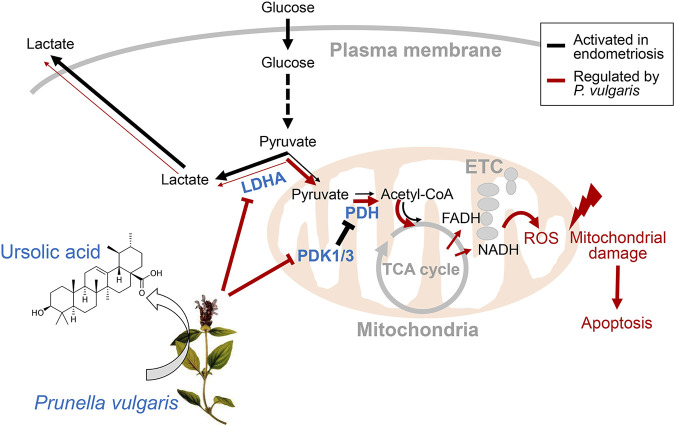
Regulation of aerobic glycolytic metabolism-mediated human endometriotic cell apoptosis by PV. In the presence of PV or ursolic acid which is one of the major bioactive component in PV, LDHA activity is promoted and PDK1/3 protein expression is suppressed. Finally, mitochondria-dependent endometriotic cell apoptosis occur, resulting from abnormally inhibited aerobic glycolytic metabolism.

Overall, we provided evidence that PV could be a novel drug candidate for preventing or treating endometriosis, and its effects are mediated by reprogramming Warburg-like metabolism. The underlying mechanism of PV-ameliorated endometriotic cell growth *in vivo* and *in vitro* is mediated via suppressed aerobic glycolytic metabolism and excessive mitoROS production. The phenomenon of PV-induced endometriotic cell apoptosis is summarized in Figure 7. As very few human studies on *P. vulgaris* have been carried out, further extensive *in vivo* and clinical trials assessing the safety and application requirements of PV for endometriosis should be carried out. Moreover, the possible effects of other active substances in PV and their combinations, followed by MOA analysis, should be studied further.

## Data Availability

The original contributions presented in the study are included in the article/Supplementary Material, further inquiries can be directed to the corresponding authors.

## References

[B1] Abraham V. C. Towne D. L. Waring J. F. Warrior U. Burns D. J. (2008). Application of a High-Content Multiparameter Cytotoxicity Assay to Prioritize Compounds Based on Toxicity Potential in Humans. J. Biomol. Screen. 13 (6), 527–537. 10.1177/1087057108318428 18566484

[B2] Andres Mde. P. Lopes L. A. Baracat E. C. Podgaec S. (2015). Dienogest in the Treatment of Endometriosis: Systematic Review. Arch. Gynecol. Obstet. 292 (3), 523–529. 10.1007/s00404-015-3681-6 25749349

[B3] Arafah M. Rashid S. Akhtar M. (2021). Endometriosis: A Comprehensive Review. Adv. Anat. Pathol. 28 (1), 30–43. 10.1097/PAP.0000000000000288 33044230

[B4] Bai Y. Xia B. Xie W. Zhou Y. Xie J. Li H. (2016). Phytochemistry and Pharmacological Activities of the Genus Prunella. Food Chem. 204, 483–496. 10.1016/j.foodchem.2016.02.047 26988527

[B5] Chapron C. Marcellin L. Borghese B. Santulli P. (2019). Rethinking Mechanisms, Diagnosis and Management of Endometriosis. Nat. Rev. Endocrinol. 15 (11), 666–682. 10.1038/s41574-019-0245-z 31488888

[B6] Chen Y. Zhu Z. Guo Q. Zhang L. Zhang X. (2012). Variation in Concentrations of Major Bioactive Compounds in Prunella Vulgaris L. Related to Plant Parts and Phenological Stages. Biol. Res. 45 (2), 171–175. 10.4067/S0716-97602012000200009 23096361

[B7] Cheung H. Y. Zhang Q. F. (2008). Enhanced Analysis of Triterpenes, Flavonoids and Phenolic Compounds in Prunella Vulgaris L. By Capillary Zone Electrophoresis with the Addition of Running Buffer Modifiers. J. Chromatogr. A. 1213 (2), 231–238. 10.1016/j.chroma.2008.10.033 18980769

[B8] Chi K. Q. Wei Z. Y. Wang K. S. Wu J. Chen W. Q. Jin X. J. (2017). Design, Synthesis, and Evaluation of Novel Ursolic Acid Derivatives as HIF-1α Inhibitors with Anticancer Potential. Bioorg. Chem. 75, 157–169. 10.1016/j.bioorg.2017.09.013 28950243

[B9] Choi H. J. Chung T. W. Choi H. J. Han J. H. Choi J. H. Kim C. H. (2018). Increased α2-6 Sialylation of Endometrial Cells Contributes to the Development of Endometriosis. Exp. Mol. Med. 50 (12), 1–12. 10.1038/s12276-018-0167-1 PMC6290765 30542051

[B10] Collins N. H. Lessey E. C. DuSell C. D. McDonnell D. P. Fowler L. Palomino W. A. (2009). Characterization of Antiestrogenic Activity of the Chinese Herb, prunella Vulgaris, Using *In Vitro* and *In Vivo* (Mouse Xenograft) Models. Biol. Reprod. 80 (2), 375–383. 10.1095/biolreprod.107.065375 18923163 PMC2746405

[B11] Cosson M. Querleu D. Donnez J. Madelenat P. Konincks P. Audebert A. (2002). Dienogest Is as Effective as Triptorelin in the Treatment of Endometriosis after Laparoscopic Surgery: Results of a Prospective, Multicenter, Randomized Study. Fertil. Steril 77 (4), 684–692. 10.1016/s0015-0282(01)03270-8 11937116

[B12] Del Rey M. J. Valín Á. Usategui A. García-Herrero C. M. Sánchez-Aragó M. Cuezva J. M. (2017). Hif-1α Knockdown Reduces Glycolytic Metabolism and Induces Cell Death of Human Synovial Fibroblasts under Normoxic Conditions. Sci. Rep. 7 (1), 3644. 10.1038/s41598-017-03921-4 28623342 PMC5473902

[B13] Doherty J. R. Cleveland J. L. (2013). Targeting Lactate Metabolism for Cancer Therapeutics. J. Clin. Invest. 123 (9), 3685–3692. 10.1172/JCI69741 23999443 PMC3754272

[B14] Dykens J. A. Jamieson J. D. Marroquin L. D. Nadanaciva S. Xu J. J. Dunn M. C. (2008). *In Vitro* assessment of Mitochondrial Dysfunction and Cytotoxicity of Nefazodone, Trazodone, and Buspirone. Toxicol. Sci. 103 (2), 335–345. 10.1093/toxsci/kfn056 18344530

[B15] Flower A. Liu J. P. Lewith G. Little P. Li Q. (2012). Chinese Herbal Medicine for Endometriosis. Cochrane Database Syst. Rev. 5, CD006568. 10.1002/14651858.CD006568.pub3 PMC12817023 22592712

[B16] Guo J. Lou M. P. Hu L. L. Zhang X. (2020). Uncovering the Pharmacological Mechanism of the Effects of the Banxia-Xiakucao Chinese Herb Pair on Sleep Disorder by a Systems Pharmacology Approach. Sci. Rep. 10 (1), 20454. 10.1038/s41598-020-77431-1 33235305 PMC7686484

[B17] Han S. Yang S. Cai Z. Pan D. Li Z. Huang Z. (2015). Anti-Warburg Effect of Rosmarinic Acid via miR-155 in Gastric Cancer Cells. Drug Des. Devel Ther. 9, 2695–2703. 10.2147/DDDT.S82342 PMC4445698 26056431

[B18] Horne A. W. Ahmad S. F. Carter R. Simitsidellis I. Greaves E. Hogg C. (2019). Repurposing Dichloroacetate for the Treatment of Women with Endometriosis. Proc. Natl. Acad. Sci. U S A. 116 (51), 25389–25391. 10.1073/pnas.1916144116 31792175 PMC6925989

[B19] Huang J. C. Ruan C. H. Tang K. Ruan K. H. (2006). Prunella Stica Inhibits the Proliferation but Not the Prostaglandin Production of Ishikawa Cells. Life Sci. 79 (5), 436–441. 10.1016/j.lfs.2006.01.018 16481008

[B20] Hwang S. M. Kim J. S. Lee Y. J. Yoon J. J. Lee S. M. Kang D. G. (2012). Anti-diabetic Atherosclerosis Effect of Prunella Vulgaris in Db/db Mice with Type 2 Diabetes. Am. J. Chin. Med. 40 (5), 937–951. 10.1142/S0192415X12500693 22928826

[B21] Jeong J. Y. Jeoung N. H. Park K. G. Lee I. K. (2012). Transcriptional Regulation of Pyruvate Dehydrogenase Kinase. Diabetes Metab. J. 36 (5), 328–335. 10.4093/dmj.2012.36.5.328 23130316 PMC3486978

[B22] Jiao X. Liu H. Lu Q. Wang Y. Zhao Y. Liu X. (2021). Study on the Mechanism of Prunella Vulgaris L on Diabetes Mellitus Complicated with Hypertension Based on Network Pharmacology and Molecular Docking Analyses. J. Diabetes Res. 2021, 9949302. 10.1155/2021/9949302 34692849 PMC8536441

[B23] Kido T. Murata H. Nishigaki A. Tsubokura H. Komiya S. Kida N. (2020). Glucose Transporter 1 Is Important for the Glycolytic Metabolism of Human Endometrial Stromal Cells in Hypoxic Environment. Heliyon 6 (6), e03985. 10.1016/j.heliyon.2020.e03985 32548315 PMC7286975

[B24] Kim B. S. Chung T. W. Choi H. J. Bae S. J. Cho H. R. Lee S. O. (2021a). Caesalpinia Sappan Induces Apoptotic Cell Death in Ectopic Endometrial 12Z Cells through Suppressing Pyruvate Dehydrogenase Kinase 1 Expression. Exp. Ther. Med. 21 (4), 357. 10.3892/etm.2021.9788 33732330 PMC7903453

[B25] Kim B. S. Chung T. W. Choi H. J. Bae S. J. Cho H. R. Lee S. O. (2021b). Caesalpinia Sappan Induces Apoptotic Cell Death in Ectopic Endometrial 12Z Cells through Suppressing Pyruvate Dehydrogenase Kinase 1 Expression. Exp. Ther. Med. 21 (4), 357. 10.3892/etm.2021.9788 33732330 PMC7903453

[B26] Kim H. I. Quan F. S. Kim J. E. Lee N. R. Kim H. J. Jo S. J. (2014). Inhibition of Estrogen Signaling through Depletion of Estrogen Receptor Alpha by Ursolic Acid and Betulinic Acid from Prunella Vulgaris Var. Lilacina. Biochem. Biophys. Res. Commun. 451 (2), 282–287. 10.1016/j.bbrc.2014.07.115 25088993

[B27] Kim J. W. Tchernyshyov I. Semenza G. L. Dang C. V. (2006). HIF-1-mediated Expression of Pyruvate Dehydrogenase Kinase: a Metabolic Switch Required for Cellular Adaptation to Hypoxia. Cell Metab 3 (3), 177–185. 10.1016/j.cmet.2006.02.002 16517405

[B28] Klemmt P. A. B. Starzinski-Powitz A. (2018). Molecular and Cellular Pathogenesis of Endometriosis. Curr. Womens Health Rev. 14 (2), 106–116. 10.2174/1573404813666170306163448 29861704 PMC5925869

[B29] Kluza J. Corazao-Rozas P. Touil Y. Jendoubi M. Maire C. Guerreschi P. (2012). Inactivation of the HIF-1α/PDK3 Signaling axis Drives Melanoma toward Mitochondrial Oxidative Metabolism and Potentiates the Therapeutic Activity of Pro-oxidants. Cancer Res. 72 (19), 5035–5047. 10.1158/0008-5472.CAN-12-0979 22865452

[B30] Koukourakis M. I. Giatromanolaki A. Sivridis E. Gatter K. C. Harris A. L. Tumor and Angiogenesis Research Group (2005). Pyruvate Dehydrogenase and Pyruvate Dehydrogenase Kinase Expression in Non Small Cell Lung Cancer and Tumor-Associated Stroma. Neoplasia 7 (1), 1–6. 10.1593/neo.04373 15736311 PMC1490315

[B31] Lee H.-C. Lin S.-C. Wu M.-H. Tsai S.-J. (2018). Induction of Pyruvate Dehydrogenase Kinase 1 by Hypoxia Alters Cellular Metabolism and Inhibits Apoptosis in Endometriotic Stromal Cells. Reprod. Sci. 26, 734–744. 10.1177/1933719118789513 30092712

[B32] Lee H. C. Lin S. C. Wu M. H. Tsai S. J. (2019). Induction of Pyruvate Dehydrogenase Kinase 1 by Hypoxia Alters Cellular Metabolism and Inhibits Apoptosis in Endometriotic Stromal Cells. Reprod. Sci. 26 (6), 734–744. 10.1177/1933719118789513 30092712

[B33] Li F. Wu Y. Chen L. Hu L. Liu X. (2019). Initial Treatment Combined with Prunella Vulgaris Reduced Prednisolone Consumption for Patients with Subacute Thyroiditis. Ann. Transl Med. 7 (3), 45. 10.21037/atm.2019.01.07 30906749 PMC6389583

[B34] Li P. Nijhawan D. Budihardjo I. Srinivasula S. M. Ahmad M. Alnemri E. S. (1997). Cytochrome C and dATP-dependent Formation of Apaf-1/caspase-9 Complex Initiates an Apoptotic Protease cascade. Cell 91 (4), 479–489. 10.1016/s0092-8674(00)80434-1 9390557

[B35] Lim G. E. Sung J. Y. Yu S. Kim Y. Shim J. Kim H. J. (2020). Pygenic Acid A (PA) Sensitizes Metastatic Breast Cancer Cells to Anoikis and Inhibits Metastasis *In Vivo* . Int. J. Mol. Sci. 21 (22). 10.3390/ijms21228444 PMC7696818 33182770

[B36] Lin W. Zheng L. Zhuang Q. Zhao J. Cao Z. Zeng J. (2013). Spica Prunellae Promotes Cancer Cell Apoptosis, Inhibits Cell Proliferation and Tumor Angiogenesis in a Mouse Model of Colorectal Cancer via Suppression of Stat3 Pathway. BMC Complement. Altern. Med. 13, 144. 10.1186/1472-6882-13-144 23800091 PMC3729539

[B37] Lin Y. Yang C. Tang J. Li C. Zhang Z. M. Xia B. H. (2020). Characterization and Anti-uterine Tumor Effect of Extract from Prunella Vulgaris L. BMC Complement. Med. Ther. 20 (1), 189. 10.1186/s12906-020-02986-5 32552673 PMC7301478

[B38] Liu Y. Gong H. Gou J. Liu X. Li Z. (2021). Dienogest as a Maintenance Treatment for Endometriosis Following Surgery: A Systematic Review and Meta-Analysis. Front. Med. 8, 652505. 10.3389/fmed.2021.652505 PMC8058209 33898487

[B39] Lum J. J. Bui T. Gruber M. Gordan J. D. DeBerardinis R. J. Covello K. L. (2007). The Transcription Factor HIF-1alpha Plays a Critical Role in the Growth Factor-dependent Regulation of Both Aerobic and Anaerobic Glycolysis. Genes Dev. 21 (9), 1037–1049. 10.1101/gad.1529107 17437992 PMC1855230

[B40] Malvezzi H. Marengo E. B. Podgaec S. Piccinato C. A. (2020). Endometriosis: Current Challenges in Modeling a Multifactorial Disease of Unknown Etiology. J. Transl Med. 18 (1), 311. 10.1186/s12967-020-02471-0 32787880 PMC7425005

[B41] Marianna S. Alessia P. Susan C. Francesca C. Angela S. Francesca C. (2017). Metabolomic Profiling and Biochemical Evaluation of the Follicular Fluid of Endometriosis Patients. Mol. Biosyst. 13 (6), 1213–1222. 10.1039/c7mb00181a 28475193

[B42] Martínez-Reyes I. Chandel N. S. (2020). Mitochondrial TCA Cycle Metabolites Control Physiology and Disease. Nat. Commun. 11 (1), 102. 10.1038/s41467-019-13668-3 31900386 PMC6941980

[B43] McKinnon B. Bertschi D. Wotzkow C. Bersinger N. A. Evers J. Mueller M. D. (2014). Glucose Transporter Expression in Eutopic Endometrial Tissue and Ectopic Endometriotic Lesions. J. Mol. Endocrinol. 52 (2), 169–179. 10.1530/JME-13-0194 24412827

[B44] Mori T. Ito F. Matsushima H. Takaoka O. Koshiba A. Tanaka Y. (2015). Dienogest Reduces HSD17β1 Expression and Activity in Endometriosis. J. Endocrinol. 225 (2), 69–76. 10.1530/JOE-15-0052 25767055

[B45] Nair A. B. Jacob S. (2016). A Simple Practice Guide for Dose Conversion between Animals and Human. J. Basic Clin. Pharm. 7 (2), 27–31. 10.4103/0976-0105.177703 27057123 PMC4804402

[B46] Nicholls D. G. (2004). Mitochondrial Membrane Potential and Aging. Aging Cell 3 (1), 35–40. 10.1111/j.1474-9728.2003.00079.x 14965354

[B47] Orazov M. R. Radzinsky V. Y. Ivanov I. I. Khamoshina M. B. Shustova V. B. (2019). Ivanov, IIOocyte Quality in Women with Infertility Associated Endometriosis. Gynecol. Endocrinol. 35 (Suppl. 1), 24–26. 10.1080/09513590.2019.1632088 31532315

[B48] Papandreou I. Cairns R. A. Fontana L. Lim A. L. Denko N. C. (2006). HIF-1 Mediates Adaptation to Hypoxia by Actively Downregulating Mitochondrial Oxygen Consumption. Cel Metab 3 (3), 187–197. 10.1016/j.cmet.2006.01.012 16517406

[B49] Park S. Jeon J. H. Min B. K. Ha C. M. Thoudam T. Park B. Y. (2018). Role of the Pyruvate Dehydrogenase Complex in Metabolic Remodeling: Differential Pyruvate Dehydrogenase Complex Functions in Metabolism. Diabetes Metab. J. 42 (4), 270–281. 10.4093/dmj.2018.0101 30136450 PMC6107359

[B50] Qiang Z. Ye Z. Hauck C. Murphy P. A. McCoy J. A. Widrlechner M. P. (2011). Permeability of Rosmarinic Acid in Prunella Vulgaris and Ursolic Acid in Salvia Officinalis Extracts across Caco-2 Cell Monolayers. J. Ethnopharmacol 137 (3), 1107–1112. 10.1016/j.jep.2011.07.037 21798330 PMC3202029

[B51] Raafat K. Wurglics M. Schubert-Zsilavecz M. (2016). Prunella Vulgaris L. Active Components and Their Hypoglycemic and Antinociceptive Effects in Alloxan-Induced Diabetic Mice. Biomed. Pharmacother. 84, 1008–1018. 10.1016/j.biopha.2016.09.095 27768926

[B52] Radzinsky V. Y. Orazov M. R. Ivanov I. I. Ivanov I. I. Khamoshina M. B. Kostin I. N. (2019). Implantation Failures in Women with Infertility Associated Endometriosis. Gynecol. Endocrinol. 35 (Suppl. 1), 27–30. 10.1080/09513590.2019.1632089 31532313

[B53] Rytkönen K. T. Heinosalo T. Mahmoudian M. Ma X. Perheentupa A. Elo L. L. (2020). Transcriptomic Responses to Hypoxia in Endometrial and Decidual Stromal Cells. Reproduction 160 (1), 39–51. 10.1530/REP-19-0615 32272449

[B54] Salah-Eldin A. E. Inoue S. Tsukamoto S. Aoi H. Tsuda M. (2003). An Association of Bcl-2 Phosphorylation and Bax Localization with Their Functions after Hyperthermia and Paclitaxel Treatment. Int. J. Cancer 103 (1), 53–60. 10.1002/ijc.10782 12455053

[B55] Semba H. Takeda N. Isagawa T. Sugiura Y. Honda K. Wake M. (2016). HIF-1α-PDK1 axis-induced Active Glycolysis Plays an Essential Role in Macrophage Migratory Capacity. Nat. Commun. 7, 11635. 10.1038/ncomms11635 27189088 PMC4873978

[B56] Shan J. Z. Xuan Y. Y. Zhang Q. Huang J. J. (2016). Ursolic Acid Sensitized colon Cancer Cells to Chemotherapy under Hypoxia by Inhibiting MDR1 through HIF-1α. J. Zhejiang Univ. Sci. B 17 (9), 672–682. 10.1631/jzus.B1600266 27604859 PMC5018614

[B57] Shen X. Yang R. An J. Zhong X. (2020). Analysis of the Molecular Mechanisms of the Effects of Prunella Vulgaris against Subacute Thyroiditis Based on Network Pharmacology. Evid. Based Complement. Alternat Med. 2020, 9810709. 10.1155/2020/9810709 33273957 PMC7676928

[B58] Stacpoole P. W. (2017). Therapeutic Targeting of the Pyruvate Dehydrogenase Complex/Pyruvate Dehydrogenase Kinase (PDC/PDK) Axis in Cancer. J. Natl. Cancer Inst. 109 (11). 10.1093/jnci/djx071 29059435

[B59] Taylor H. S. Kotlyar A. M. Flores V. A. (2021). Endometriosis Is a Chronic Systemic Disease: Clinical Challenges and Novel Innovations. Lancet 397 (10276), 839–852. 10.1016/S0140-6736(21)00389-5 33640070

[B60] Uludag S. Z. Demirtas E. Sahin Y. Aygen E. M. (2021). Dienogest Reduces Endometrioma Volume and Endometriosis-Related Pain Symptoms. J. Obstet. Gynaecol. 41 (8), 1246–1251. 10.1080/01443615.2020.1867962 33629621

[B61] Urbańska K. Orzechowski A. (2019). Unappreciated Role of LDHA and LDHB to Control Apoptosis and Autophagy in Tumor Cells. Ijms 20 (9), 2085. 10.3390/ijms20092085 31035592 PMC6539221

[B62] Wang P. Li Z. Fu L. Zhu J. Wu X. Wang Z. (2014). Effects of Extracts of Prunella Vulgaris L. On Proteome of Human Lung Adenocarcinoma Cell Line A549. Zhonghua Yi Xue Za Zhi 94 (28), 2216–2221. 25331476

[B63] Wang S. J. Wang X. H. Dai Y. Y. Ma M. H. Rahman K. Nian H. (2019). Prunella Vulgaris: A Comprehensive Review of Chemical Constituents, Pharmacological Effects and Clinical Applications. Curr. Pharm. Des. 25 (3), 359–369. 10.2174/1381612825666190313121608 30864498

[B64] Wang W. J. Sui H. Qi C. Li Q. Zhang J. Wu S. F. (2016). Ursolic Acid Inhibits Proliferation and Reverses Drug Resistance of Ovarian Cancer Stem Cells by Downregulating ABCG2 through Suppressing the Expression of Hypoxia-Inducible Factor-1α In vitro. Oncol. Rep. 36 (1), 428–440. 10.3892/or.2016.4813 27221674

[B65] Xiang D. Zhao M. Cai X. Wang Y. Zhang L. Yao H. (2020). Betulinic Acid Inhibits Endometriosis through Suppression of Estrogen Receptor β Signaling Pathway. Front. Endocrinol. 11, 604648. 10.3389/fendo.2020.604648 PMC7759155 33362719

[B66] Yang K. Guo K. Q. Wu H. Y. Ye L. X. Xia H. (2007). Clinical Effect of Prunrllae Oral Solution in Treating Hyperthyrea. Zhongguo Zhong Yao Za Zhi 32 (16), 1706–1708. 18027674

[B67] Young V. J. Ahmad S. F. Brown J. K. Duncan W. C. Horne A. W. (2016). ID2 Mediates the Transforming Growth Factor-Β1-Induced Warburg-like Effect Seen in the Peritoneum of Women with Endometriosis. Mol. Hum. Reprod. 22 (9), 648–654. 10.1093/molehr/gaw045 27385728

[B68] Young V. J. Brown J. K. Maybin J. Saunders P. T. Duncan W. C. Horne A. W. (2014). Transforming Growth Factor-β Induced Warburg-like Metabolic Reprogramming May Underpin the Development of Peritoneal Endometriosis. J. Clin. Endocrinol. Metab. 99 (9), 3450–3459. 10.1210/jc.2014-1026 24796928 PMC4207934

[B69] Yu F. Zhang L. Ma R. Liu C. Wang Q. Yin D. (2021). The Antitumour Effect of Prunella Vulgaris Extract on Thyroid Cancer Cells *In Vitro* and *In Vivo* . Evid. Based Complement. Alternat Med. 2021, 8869323. 10.1155/2021/8869323 33505511 PMC7811421

[B70] Zhang W. Zhang S. L. Hu X. Tam K. Y. (2015). Targeting Tumor Metabolism for Cancer Treatment: Is Pyruvate Dehydrogenase Kinases (PDKs) a Viable Anticancer Target? Int. J. Biol. Sci. 11 (12), 1390–1400. 10.7150/ijbs.13325 26681918 PMC4671996

[B71] Zhang Y. Ma K. Sadana P. Chowdhury F. Gaillard S. Wang F. (2006). Estrogen-related Receptors Stimulate Pyruvate Dehydrogenase Kinase Isoform 4 Gene Expression. J. Biol. Chem. 281 (52), 39897–39906. 10.1074/jbc.M608657200 17079227

[B72] Zhang Z. Zhou Y. Lin Y. Li Y. Xia B. Lin L. (2020). GC-MS-based Metabolomics Research on the Anti-hyperlipidaemic Activity of Prunella Vulgaris L. Polysaccharides. Int. J. Biol. Macromol 159, 461–473. 10.1016/j.ijbiomac.2020.05.003 32387363

[B73] Zhao J. Ji D. Zhai X. Zhang L. Luo X. Fu X. (2018). Oral Administration of Prunella Vulgaris L Improves the Effect of Taxane on Preventing the Progression of Breast Cancer and Reduces its Side Effects. Front. Pharmacol. 9, 806. 10.3389/fphar.2018.00806 30123125 PMC6085460

[B74] Zheng J. Dai Y. Lin X. Huang Q. Shi L. Jin X. (2021). Hypoxia-induced L-actate D-ehydrogenase A P-rotects C-ells from A-poptosis in E-ndometriosis. Mol. Med. Rep. 24 (3). 10.3892/mmr.2021.12276 PMC8281285 34278456

[B75] Zubrzycka A. Zubrzycki M. Perdas E. Zubrzycka M. (2020). Genetic, Epigenetic, and Steroidogenic Modulation Mechanisms in Endometriosis. J. Clin. Med. 9 (5). 10.3390/jcm9051309 PMC7291215 32370117

