# Corrigendum: Water-extracted *Prunella vulgaris* alleviates endometriosis by reducing aerobic glycolysis

**DOI:** 10.3389/fphar.2022.1112004

**Published:** 2022-12-13

**Authors:** Min Kyoung Cho, Ling Jin, Jung Ho Han, Jung-Suk Jin, Se-Yun Cheon, Su Shin, Sung-Jin Bae, JangKyung Park, Ki-Tae Ha

**Affiliations:** ^1^ Korean Medicine Research Center for Healthy Aging Pusan National University, Yangsan, South Korea; ^2^ Department of Korean Medical Science School of Korean Medicine Pusan National University, Yangsan, South Korea; ^3^ Department of Anatomy Kosin University College of Medicine, Busan, South Korea; ^4^ Department of Korean Obstetrics and Gynecology Pusan National University Korean Medicine Hospital, Yangsan, South Korea

**Keywords:** Aerobic glycolysis, endometriosis, lactate dehydrogenase, pyruvate dehydrogenase kinase, Prunella vulgaris, ulsoric acid, Warburg-like metabolism

In the published article, there was an error in the **Funding** statement. The grant number pertaining to the Korea Health Technology R&D Project was incorrectly quoted. The corrected statement appears below.

**“**This research was supported by a National Research Foundation of Korea (NRF) grant funded by the Korean government (MIST) (No. NRF-2019R1G1A1100022) and by a grant of the Korea Health Technology R&D Project through the Korea Health Industry Development Institute (KHIDI), funded by the Ministry of Health and Welfare, Republic of Korea (grant no. HF20C0055).”

The authors apologize for this error and state that this does not change the scientific conclusions of the article in any way. The original article has been updated.

