# Supplementary material for: Water-Extracted Prunella vulgaris Alleviates Endometriosis by Reducing Aerobic Glycolysis
Source: Front Pharmacol. 2022 Apr 4;13:872810. doi: 10.3389/fphar.2022.872810 (PMC9014096; doi:10.3389/fphar.2022.872810)
Supplement: Supplementary file 1 [file DataSheet1.pdf]

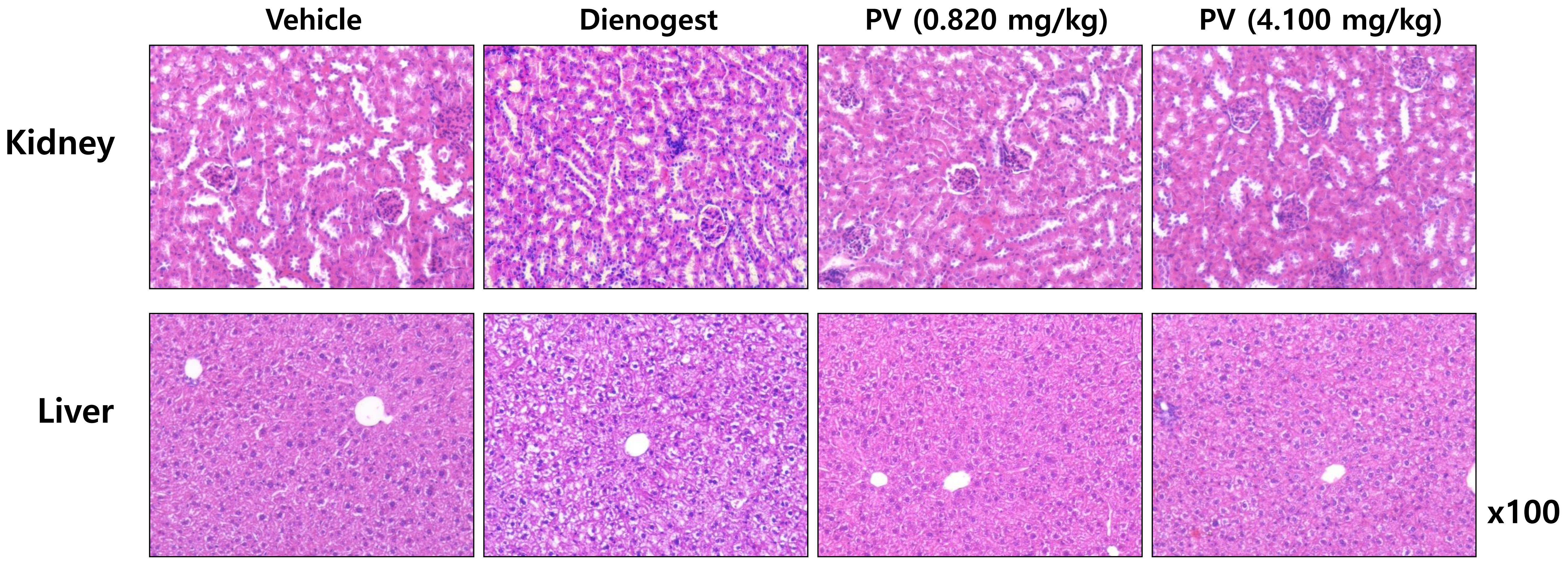

Supplementary Figure S1. Hematoxylin and eosin-stained kidney and liver sections from mice after endometriosis induction (x100)

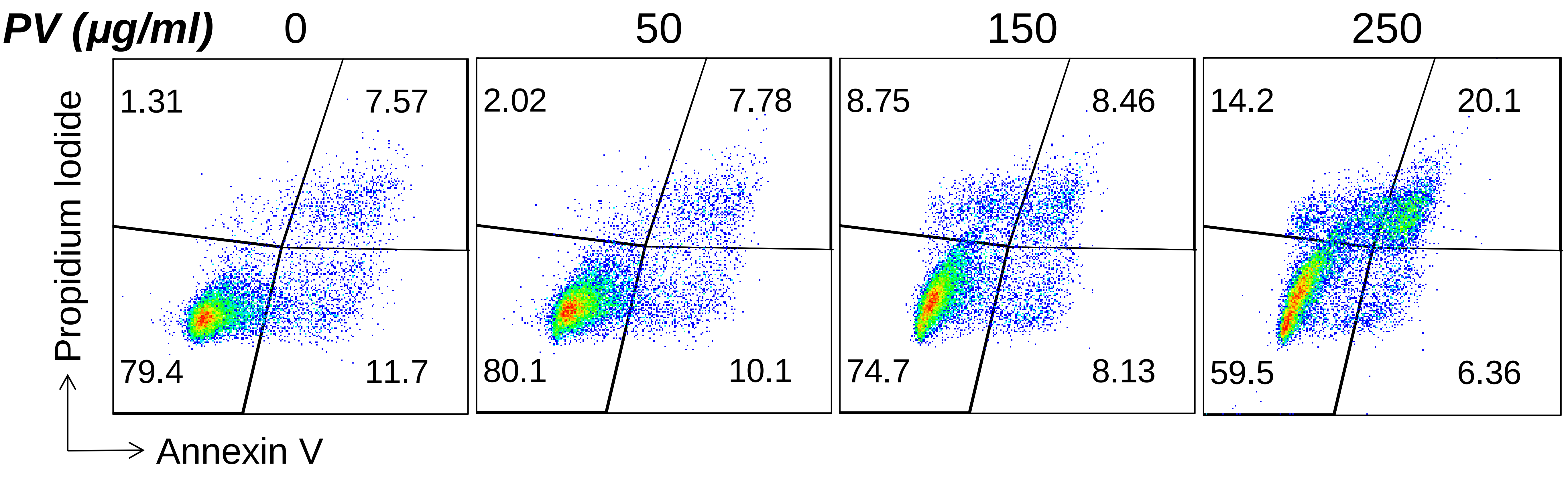

Supplementary Figure S2. Effect of PV on the apoptosis of 12Z cells. 12Z cells were treated with increasing concentrations of PV for 24 h. The apoptosis of 12Z cells was analyzed by flow cytometry after PI and Annexin V staining.

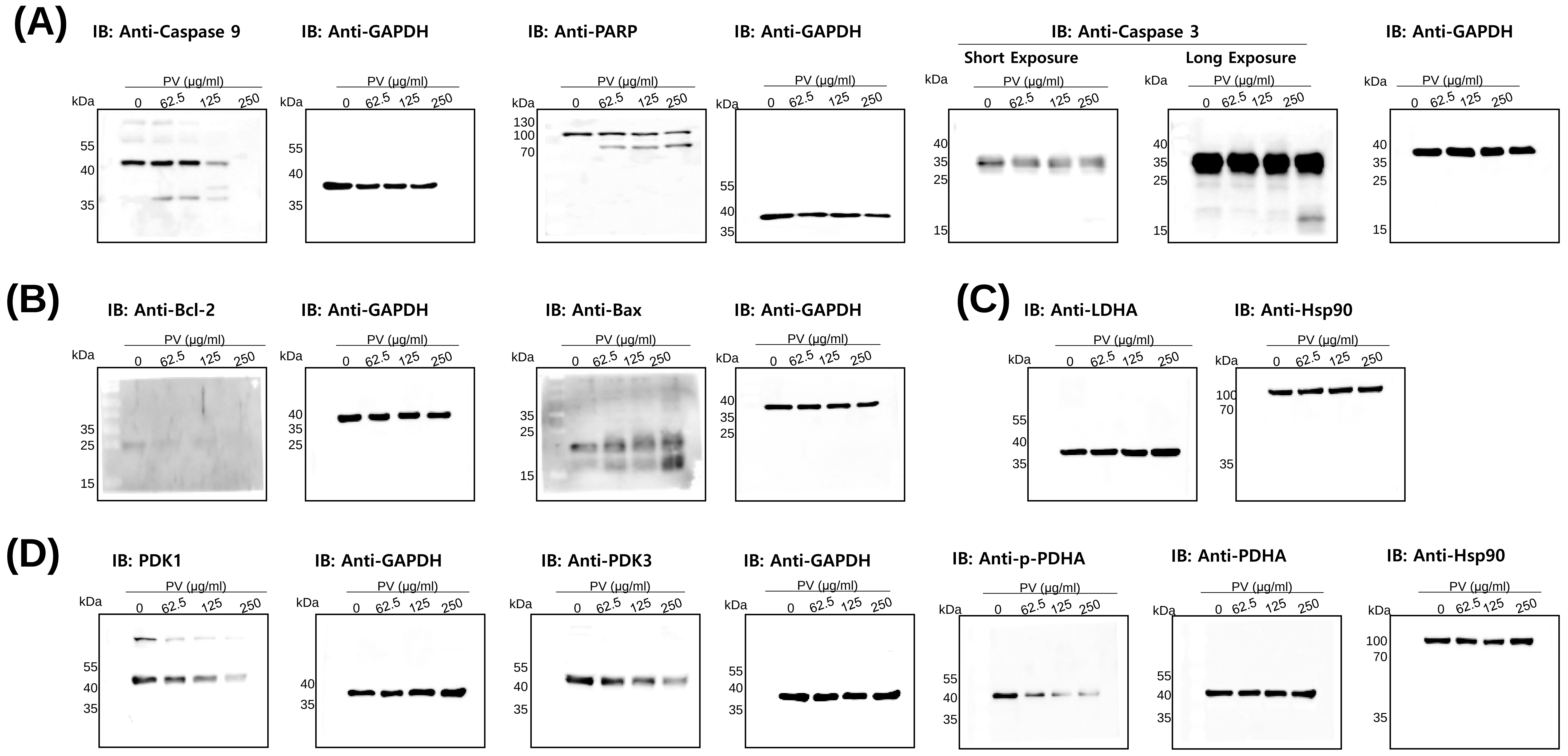

Supplementary Figure S3. The whole blot used in this study. (A), Figure 2C; (B), Figure 3C; (C), Figure 4C; (D), Figure 4D
